# Topical Glucocorticoid Use and the Risk of Posttransplant Diabetes

**DOI:** 10.1155/2023/3648178

**Published:** 2023-01-21

**Authors:** Simon Bøtker, Henrik Birn, Lara Aygen Øzbay

**Affiliations:** ^1^Department of Nephrology, Palle Juul-Jensens Blvd 99, Aarhus University Hospital, Aarhus 8000, Denmark; ^2^Faculty of Health, Aarhus University, Aarhus, Denmark

## Abstract

Systemically administered glucocorticoids constitute an essential part of the immunosuppressive regimen for transplant recipients, yet their known risks of causing hyperglycemia or posttransplant diabetes require close monitoring and minimisation of use, when possible, to prevent detrimental effects on patient morbidity and graft survival. Topical glucocorticoids, on the other hand, are rarely considered to affect glucose metabolism and therefore seldomly monitored, despite their wide and in some cases, long-term use. We report a case of a renal transplant recipient presenting with acute hyperosmolar hyperglycemia after treatment with topical glucocorticoids and present a mini review of the literature.

## 1. Introduction

Renal transplant recipients are known to have an increased risk of posttransplant diabetes (PTDM) given their predispositions, among which insulin resistance, immunosuppressive medications, and traditional risk factors for type 2 diabetes (T2D) play a major role [1, 2]. PTDM develops in 10–20% of renal transplant recipients [1] and may impact both short- and long-term outcomes of renal transplant recipients negatively in terms of cardiovascular morbidity, graft function, and overall patient survival [3–6]. This has led to intense efforts, studies, and guidelines throughout the past decade on strategies to prevent PTDM [7–12], with particular interest in steroid-sparing regimes. Although a recent retrospective analysis with a long-termfollow-up of 15 years found that rapid glucocorticoid discontinuation was associated with reduced onset of PTDM [13], a Cochrane systematic review from 2017 and a meta-analysis from 2010 found no benefit of glucocorticoid withdrawal on PTDM and also asserted an increased risk of acute rejection [14, 15]. To avoid rejection and risk of graft dysfunction, many transplant centers maintain glucocorticoids as part of a complete immunosuppressive regime.

Topical glucocorticoids (TS) are frequently used for a wide range of dermatological conditions and are considered relatively safe [16]; however, systemic absorption and side effects such as hyperglycemia, osteoporosis, and glaucoma have been reported and are mentioned in the summary of product characteristics [17, 18]. Significant associations between TS and the risk of T2D have been shown in previous, retrospective studies [19–21]. The magnitude of this risk remains uncertain, and only a few case reports have described the use of TS resulting in overt T2D [17, 22–25].

The risk may be dependent on dose, duration, and patient-related factors such as specific skin disease, BMI, concurrent diseases, and medication. The clinical practice of reporting systemic side effects of TS is poor, and considerations of alternative treatments to TS or screening for T2D during its use are not routinely performed at all dermatology clinics or by general practitioners [17].

Hence, the diabetogenicity of TS in patients with an underlying risk of diabetes, such as renal transplant recipients, warrants further investigation. We herein describe the first case of a renal transplant recipient who developed acute hyperosmolar nonketotic hyperglycemia and PTDM after treatment with TS and provide a mini review of the literature.

## 2. Case Presentation

The patient was a 58-year-old Caucasian male without a family history of T2D who received an ABO-compatible living donor renal transplant following renal failure and nephrotic syndrome due to hereditary ApoA-1 amyloidosis. SAP-scintigraphy revealed additional amyloid deposition in the liver and spleen. Other comorbidities included hypertension, hypercholesterolemia, and gout, which were well treated.

He was maintained on standard triple immunosuppressive therapy with tacrolimus, mycophenolate, and low-dose oral prednisolone (5 mg q.d.), without any complications or episodes of acute rejection. Six years after transplantation, a gradual decline in renal graft function was observed, and a renal graft biopsy showed evidence of recurrent amyloidosis.

**Figure 1:**
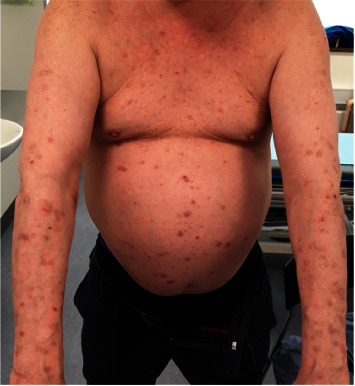
Picture of the patient's skin lesions before occlusive therapy.

Seven years after transplantation, he was diagnosed with biopsy-proven prurigo nodularis and treated with TS in the form of clobetasol propionate (0.5 mg/g) q.d. for three weeks and every second day for the following two months. Due to persistent disease, treatment was intensified by using daily 3-hour occlusional TS on the entire body except the face for two weeks. The cumulative dose of topical clobetasol propionate was 730 g and the cumulative dose of systemic glucocorticoids at this time was 10.435 mg (105 mg/kg). The skin lesions are shown in Figure 1.

Prior to TS-treatment, random glucose levels had been continuously normal for several years, ranging from 4.9–8.7 mmol/L, yet a HbA1c of 46 mmol/mol was noted 5 years earlier, suggesting a possible prediabetic condition. Within two weeks of occlusional TS-treatment, the patient developed increased thirst, polyuria, and a weight loss of 5 kg and presented seventeen days after initiation of treatment with hyperosmolar nonketoacidosis due to hyperglycemia and a blood glucose level of 46.9 mmol/L. Three months prior to this event, he had a random glucose level of 8.7 mmol/L. He weighed 99.6 kg (BMI of 31.8 kg/m^2^). The renal graft function was stable with an eGFR of 21 ml/min/1.73 m^2^. He was admitted to hospital, immediately discontinued on TS, and started daily subcutaneous injections with short-acting insulin aspart, requiring 32 units per day, and insulin glargine injections at an initial dose of 16 units once daily. After a few days, he was discharged with 30 units of insulin glargine once daily and oral linagliptin 0.6 mg q.d. Short-acting insulin was prescribed with meals as necessary. He consulted his dermatologist afterwards and restarted TS at a reduced dose daily for two weeks, followed by twice per week. 20 days later, at an outpatient follow-up, his fasting glucose had normalized at 7.6 mmol/L, and his HbA1c levels had declined from 93 mmol/mol to 76 mmol/mol. The anti-diabetic treatment consisted of insulin glargine 20 units once daily and liraglutide 0.6 mg q.d. Short-acting insulin was no longer required.

Current status of the patient approximately 15 months after diagnosis of PTDM is improved glycemic control with HbA1c levels of 45 mmol/mol on insulin glargine 16 units q.d. and liraglutide 1.2 mg q.d. The skin condition has improved on alternative treatment with dupilumab and pimecrolimus. He is no longer treated with TS.

## 3. Discussion

The present study was performed according to PRISMA guidelines and recommendations [26].

Searches were carried out using the PubMed, Scopus, Web of Science, and Cochrane Library databases. The search strategy and results are summarized in Table 1. More searches were made without the inclusion of any main articles. During searches, different article types were selected to narrow the search. The incorporated advanced search builder was applied using a combination of relevant MeSH-terms and free text keywords separated by “AND” or “OR.” The articles needed to match the two following criteria:

1. Hyperglycemia, diabetes mellitus (DM), T2D, insulin resistance, PTDM, or new-onset DM after transplantation
2. TS-treatment

Identical searches were made using each of the databases, though without identifying any articles beyond those identified using PubMed.

We included all relevant articles in English or Danish regardless of study design. Articles not addressing this specific topic as well as nonhuman studies were excluded.

Extended searches of relevant articles using the “similar articles,” “cited by,” and “reference” lists were performed using the before mentioned databases.

We identified 158 potentially relevant publications from the electronic searches as well as the extended sources including reference lists. Abstracts from these were screened, and after excluding duplicate or irrelevant references, 78 potential articles were retrieved. After detailed evaluation, one meta-analysis, two review articles, four case-control studies from three articles, and four case reports were included in this review.

To our knowledge, this report describes the first case of hyperosmolar nonketotic hyperglycemia and PTDM in a renal transplant recipient induced by TS-treatment.

TS are widely used to treat skin conditions, however, the impact of systemic absorption on glucose metabolism and subsequent T2D is unclear. While TS generally are of a molecular weight allowing systemic absorption [28, 29], the risk of systemic absorption is only briefly mentioned in the summary of product characteristics, and hyperglycemia is solely mentioned in relation to Cushing syndrome [30].

A recent meta-analysis of four case-control studies including more than 350.000 subjects found a statistically significant association between TS-use and T2D-development with an odds ratio (OR) of 1.24 (95% CI 1.15–1.34) [21]. It concluded that prolonged use of TS leads to higher cumulative dose and increased risk of T2D irrespective of the potency of TS. The analysis, however, was limited by heterogeneity within the databases and between the studies in terms of what defines prolonged TS duration. Among the included studies, the study by Andersen et al. [19] was the only one to demonstrate a tendency towards a dose-response relationship between higher TS-potency and risk of T2D. Consequently, the authors recommended the use of alternative treatments to potentially diabetogenic high-potency TS. The study was based on high-quality databases including both case-control and cohort studies, providing a large number of subjects and information on confounding comorbidities with the exclusion of diseases related to insulin resistance and DM. However, unlike orally administered glucocorticoids, the absorption rate of TS differs depending on the administration area and the condition of the layers of the skin [23, 29]. The authors were unable to differentiate between these factors, resulting in potential bias. Likewise, prolonged use may result in higher cumulative systemic doses, as could occlusion therapy due to weakness of the stratum corneum, regardless of glucocorticoid potency [17, 21, 22]. Furthermore, TS are often used intermittently in practice, and healthcare data do not capture medication compliance, so exposure definitions may not fully reflect actual use. In a Dutch study by Van der Linden et al., the cumulative dose of TS measured in a four-year period before the study's index date was measured. The cumulative dose was associated with an increased risk of DM with doses of 181–365 mg being associated with an OR's of 1.3 (95% CI 1.09–1.49), 366–730 mg of 1.3 (95% CI 1.06–1.47), and 731–1460 mg of 1.4 (95% CI 1.15–1.66) and a significant test for trend (*P* < 0.02) [20]. TS are not available as over-the-counter medicine in the Netherlands, minimizing the potential bias from inaccurate recording of exposure. Contrary to these studies, Gulliford et al. did not find any association between TS and DM, albeit there were several limitations such as imprecise exposure and outcome classification, as well as relatively low TS doses possibly underestimating a true effect [27].

Only a few cases of new-onset DM following TS-treatment have been published [22–25]. Kahara et al. reported a patient receiving class VII (lowest potency) TS applied to the oral mucosa for several months to treat lichen planus. The patient developed new-onset diabetes requiring the initiation of oral antidiabetic medication [23]. Sue and Milanesi described a patient with known type 1 DM admitted with acute hyperglycemia and required five times his typical daily insulin dose after only two days of occlusive treatment with class I TS on a large area of his body for psoriasis [22], similar to our case. The development of acute hyperosmolar nonketotic hyperglycemia and PTDM in our patient was most likely caused by the occlusive TS-therapy given the severe presentation and temporal relationship. However, he had several additional risk factors that may have led to a prediabetic state for many years prior to this event. These include his immunosuppressive regime, hereditary amyloidosis, and a BMI > 30. Hereditary ApoA1 amyloidosis involving the pancreas or resulting in T2D has never been described previously [31], and SAP scintigraphy in our patient showed no amyloid depositions in his pancreas. However, amyloid depositions in the liver could potentially impair hepatic insulin sensitivity, and ApoA1 has been shown to modulate insulin secretion in in vitro studies [32], both of which have been linked to prediabetes and T2D. The development of PTDM may have increased the risk of graft function deterioration as well as future adverse cardiovascular outcomes.

## 4. Conclusion

In conclusion, the presented case with TS-induced severe hyperglycemia and PTDM in a renal transplant recipient is in line with retrospective analyses supporting the diabetogenic effect of TS. Although the risk may be less than for systemically administered glucocorticoids, physicians treating patients with prolonged, high potency, or occlusive TS therapy should be aware of possible systemic side effects and screen/monitor for hyperglycemia, especially when the diabetes risk is already increased. The development of PTDM in a renal transplant recipient is associated with an increased risk of cardiovascular disease, accelerated loss of graft, and increased mortality and should be prevented whenever possible.

## Figures and Tables

**Table 1:** Systematic search in PubMed.

Search number	Search words	Comments	Hits	Abstracts read	Full articles read	Articles included	
1	(Topical steroids) AND (diabetes mellitus)		313	67	33	3	Kahara et al. [23], Phan and Smith [21], and Hongo et al. [24]
2	(Topical corticosteroid) AND (hyperglycemia)	Search with lack of sensitivity	22	8	8	3	Hengge et al. [17], Sue and Milanesi [22], and Andersen et al. [19]
3	(Topical) AND “adrenal cortex hormones/adverse effects”(MESH)	Many reviews about TS selection and GC withdrawal	1.500	43	23	2	Callen et al. [16] and van der Linden et al. [20]
4	Diabetes AND “glucocorticoids/adverse effects”(MESH)	Many reviews about systemic GC treatment and risk of DM	861	35	11	1	Gulliford et al. [27]
5	“Topical corticosteroid” AND “iatrogenic”		12	5	3	1	Ahmed et al. [25]

## Data Availability

All data used to describe the case report and perform the literature review are available upon reasonable request to the corresponding author.
